# Intermittent Hypoxic Training at Lactate Threshold Intensity Improves Aiming Performance in Well-Trained Biathletes with Little Change of Cardiovascular Variables

**DOI:** 10.1155/2019/1287506

**Published:** 2019-09-25

**Authors:** Miłosz Czuba, Grzegorz Bril, Kamila Płoszczyca, Zofia Piotrowicz, Małgorzata Chalimoniuk, Robert Roczniok, Agnieszka Zembroń-Łacny, Dagmara Gerasimuk, Józef Langfort

**Affiliations:** ^1^Department of Kinesiology, Institute of Sport, Warsaw, Poland; ^2^Department of Sports Training, The Jerzy Kukuczka Academy of Physical Education in Katowice, Faculty of Physical Education, Katowice, Poland; ^3^Faculty of Medicine and Health Sciences, University of Zielona Góra, Zielona Góra, Poland; ^4^Department of Tourism and Health in Biala Podlaska, Józef Piłsudski University of Physical Education in Warsaw, Warsaw, Poland

## Abstract

The main objective of this research was to evaluate the efficacy of intermittent hypoxic training (IHT) on aiming performance and aerobic capacity in biathletes. Fourteen male biathletes were randomly divided into a hypoxia group (H) (*n* = 7), which trained three times per week in a normobaric hypoxic environment (FiO_2_ = 16.5%, 2000 m a.s.l.) with lactate threshold intensity (LT) determined in hypoxia, and a control group (C) (*n* = 7), which exercised under normoxic conditions with LT intensity determined in normoxia. The training program included three weekly microcycles, followed by three days of recovery. The main part of the interval workout consisted of four 7 min (1^st^ week), 8 min (2^nd^ week), or 9 min (3^rd^ week) running bouts at treadmill separated by 2 minutes of active recovery. After the warm-up and during the rest between the bouts, the athletes performed aiming to the target in the standing position with a sporting rifle (20 s). The results showed that the IHT caused a significant (*p* < 0.05) increase in retention time in the target at rest (RT9_rest_) by 14.4% in hypoxia, whereas RT postincremental test (RT9_post_) increased by 27.4% in normoxia and 26.7% in hypoxia. No significant changes in this variable were found in group C. Additionally, the capillary oxygen saturation at the end of the maximal effort (SO_2capillary max_) in hypoxia increased significantly (*p* < 0.001) by ∼4% after IHT. The maximal workload during the incremental test (WR_max_) in normoxia also increased significantly (*p* < 0.001) by 6.3% after IHT. Furthermore, in absolute and relative values of VO_2max_ in normoxia, there was a propensity (*p* < 0.07) for increasing this value by 5% in group H. In conclusion, the main findings of this study showed a significant improvement in resting and postexercise aiming performance in normoxia and hypoxia. Furthermore, the results demonstrated beneficial effects of the IHT protocol on aerobic capacity of biathletes.

## 1. Introduction

Biathlon is a winter sport that combines cross-country skiing with rifle shooting. Biathlon competitions consist of 3–5 skiing laps incorporating 2–4 shooting rounds which comprise 5 shots fired from the prone or standing positions. A penalty lap or penalty time is given for each failed target, and such adverse events strongly influence the biathletes' final competition result. The shooting trials are very challenging for biathletes because they demand coordination of visual input, postural balance, and precise timing of index finger contraction on the trigger [1, 2] and usually take place in challenging environmental conditions including cold and altitude. In the latter case, low (500–1500 m a.s.l.) to moderate (1500–3000 m a.s.l.) altitudes are frequently used for completions as well as for biathletes' training [3]. Despite the mentioned difficulties, the cross-country skiing is performed under conditions of intense cardiovascular load reaching 90% of maximum heart rate [4]. It implies that shooting trials, especially the last few, may take part under central [5–7] and/or peripheral fatigue and each of them can be exaggerated by the hypoxia-induced deteriorating effect on muscles and the central nervous system [8–10]. However, some data show that peripheral and central neuromuscular adaptations during a sustained fatiguing contraction are similar regardless of whether the contraction is performed in a normoxic environment or in acute hypoxia [11].

Previous research has shown that each reduction by 1% in oxygen saturation of the blood (SO_2_) below the level of 95% results in a decrease in VO_2max_ by 1-2% [12]. The decline in SO_2_ occurs already for low- and medium-intensity exercise, even in well-trained elite athletes [13]—a phenomenon shown to impair exercise performance [14]. Interestingly, previous findings indicate that exercise intensity has a negligible effect on shooting in the prone position but strongly affects shooting in a standing position by altering the stability of the hold [15].

In the case of biathlon, hypoxia is an inherent environmental stimulus in motor preparation of athletes because of the need for training on trails covered with snow in the preparation period (summer and fall) that forces athletes to move to the altitude of 2000 to 3000 m above sea level. The training program most commonly used altitude by biathletes is based on the classic “live high, train high” (LH-TH) method. Its modification which combines both aerobic and interval training programs (live high, base train high, interval train low—HiHiLo) revealed a significant improvement in VO_2max_ by 3% and in the 3 km race time by 5.8 s on average [16]. When this method was applied to biathletes at 2000 m a.s.l., an improvement in hematological indices was observed without any changes in VO_2max_. However, these changes were accompanied by reduced energy expenditure [3]. The available reports demonstrated that the training process performed in the hypoxic environment can decrease the energy cost of exercise by reducing oxygen demand by 3–10% [3, 17]. Additionally, previous results concerning the utilization of hypoxic methods for improvement in exercise performance in biathlon and other endurance sports revealed their favorable impact on blood oxygen-carrying capacity and/or exercise capacities in normoxia [3, 18–21]. For example, in our previous study [3], we found a significant 5-6% increase in hemoglobin concentration, number of erythrocytes, and hematocrit value and a 16% increase in the percentage of reticulocytes in elite biathletes after 3 weeks of HiHiLo procedure at 2000 m altitude. Furthermore, Wehrlin et al. [20] demonstrated a 4% increase in VO_2max_ and improvement of 5000 m running time (∼2%) after 24-day LH-TL at 2500 m, accompanied by the improvement of hemoglobin mass and red cell volume by 5% in orienteers.

However, another important issue for biathletes that may influence sport performance is the effect of hypoxia on shooting efficiency. This particular issue has not previously been addressed in the literature. Studies conducted on representatives of other sports have found that exposure to moderate hypoxia limits cognitive abilities and ability to perform psychomotor activities [22]. Likewise, the most recent examination performed with rifles by untrained individuals confirmed the impairment of marksmanship at higher simulated altitudes, both at rest and postexercise [23]. The impairment of shooting efficiency in a hypoxic environment seems to be especially important in biathlon because both training and competition are held at altitude.

There is some evidence that a novel concept applied in preparation of athletes for competitions based on hypoxia-exercise integration has better potential to induce optimal training adaptations at physiological, biochemical, and genetic levels [24]. It is considered that intermittent hypoxic training (IHT) can act as an additional stimulus for the muscle tissue adaptations and may be useful for preparing for competitions at altitude [25]. However, further evidence is needed to explain how hypoxia may modify biathletes' shooting performance. Thus, the present study verifies the hypothesis if IHT at lactate threshold with implemented aiming exercise may simultaneously improve aerobic capacity and aiming performance of well-trained biathletes. Based on findings published by Moore et al. [23] and Kryskow et al. [26], one might presume that the primary mechanism behind the improvement of shooting abilities after adaptation to hypoxia can be an increase in SO_2_. To test this assumption, we integrated IHT in the preparation period to reveal the impact of normobaric hypoxia on aiming performance of biathletes either in hypoxic or in normoxic environments. Furthermore, cardiorespiratory (i.e., maximal oxygen uptake, maximal ventilation, maximal respiratory ratio, and maximal heart rate during the incremental test) variables were also measured pre- and post-IHT.

## 2. Materials and Methods

### 2.1. Participants

This study examined 14 well-trained male biathletes. In order to detect a small effect size (*d* = 0.4) [27, 28], with a power of 0.80 and *α* = 0.05, a sample size of at least 12 individuals was determined using the GPower 3.1 software. We recruited the number of participants that was nearly 20% higher than the necessary level, taking into account the potential chance events (illness, injury, etc.). The basic inclusion criteria were a minimum of five years of training experience and at least 4 months of rest from previous altitude training. Characteristics of the participants are reported in Table 1.

All participants were randomly divided into a hypoxia (H) group (*n* = 7), who trained in a normobaric hypoxic environment, and a control (C) group (*n* = 7), which exercised under normoxic conditions. In the H group, the concept of IHT procedure was proposed, according to our previous study [29].

Participants were allocated to conditions using a computer-generated randomized list (block randomization: 7 blocks of 2 participants, each person within a block was assigned to one of the two conditions) [30]. All athletes had current medical examinations, without any contraindications to performing exhaustive exercise in a hypoxic environment. The participants provided their written, voluntary, informed consent before participation. No adverse effects in both experimental and control groups were observed during the experiment. There were also no dropouts from the study.

The research project was conducted according to the Helsinki Declaration and was approved (no. 5/2013 and 10/2015) by the Ethics Committee for Scientific Research at the Jerzy Kukuczka Academy of Physical Education in Katowice, Poland.

### 2.2. Experimental Design

The research was conducted at the beginning of the preparatory period. The experiment was divided into three series of tests performed in a laboratory environment. All participants were familiarized with the test protocol one week before the first evaluations. Then, three microcycles (three weeks) with a progressive training load were applied, followed by a short recovery microcycle (three days). The final evaluations were performed after the recovery microcycle. The testing procedures in all series were identical for all participants. Participants in both groups were not informed about environmental conditions of the testing protocol.

### 2.3. Testing Protocol

**Figure 1:**
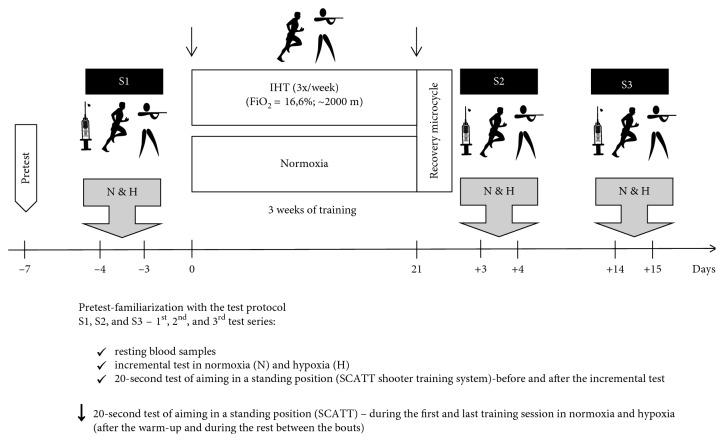
Illustration of the study design.

This research project used three test series (S1, S2, and S3). The first test was performed 4 and 3 days before training (S1), the second test at 3 and 4 days after training (S2), and the third test at 14 and 15 days after training (S3). All test series (S1, S2, and S3) included one day of examinations in normoxia and one day in normobaric hypoxia (2000 m). The details of the study design are presented in Figure 1.

On the first day of each test series (S1, S2, and S3), before breakfast and after an overnight fast, body mass and body composition were evaluated using the electrical impedance technique (Inbody 720, Biospace Co., Japan). Two hours after a light breakfast (5 kcal/1 kg of body weight, 50% carbohydrates, 20% proteins, and 30% fats), the incremental treadmill test was performed to determine aerobic capacity.

The incremental running test started with a speed of 6 km/h that was increased by 2 km/h every 3 minutes until the speed reached 14 km/h. Exercise intensity was then increased by adjusting the treadmill incline by 2.5% every 3 minutes to volitional exhaustion. The exercise intensity was expressed in watts (W), as calculated by the MetaSoft software (Cortex, Germany). If a participant terminated the test before completing a given workload, the maximum workload was calculated from the formula WR_max_ = WR_k_ + (*t*/*T* × WR_p_) [31], where WR_k_ = previous workload, *t* = exercise duration with the workload until premature failure, *T* = duration of each workload, and WR_p_ = amount of workload by which exercise intensity increased during the test.

During the test, heart rate (HR), oxygen uptake (VO_2_), expired carbon dioxide (CO_2_), and minute ventilation (VE) were measured continuously using the MetaMax 3B telemetry spiroergometer (Cortex, Germany) in the breath-by-breath mode. The criteria of reaching VO_2max_ included the following: a plateau in the level of VO_2_ or a gradual decrease in peak VO_2_ during the maximal workload, respiratory exchange ratio (RER above 1.1), blood lactate concentration (LA above 8.0 mmol/l), as well as predicted maximal heart rate [32]. All athletes finished the incremental test with required criteria of reaching VO_2max_.

Fingertip capillary blood samples for the assessment of lactate (LA) concentration (Biosen C-line Clinic, EKF-diagnostic GmbH, Germany) were drawn at rest and at the end of each step of the test, as well as during the 3^rd^, 6^th^, 9^th^, and 12^th^ minute of recovery. The lactate threshold was determined by the D-max method [33]. Our earlier study [34] demonstrated that LT determined by the D-max method corresponds to the maximal lactate steady state (MLSS). Additionally, capillary rest and postexercise blood samples were used to determine acid-base equilibrium and capillary oxygen saturation of hemoglobin (SO_2capillary_) (RapidLab 248, Bayer Diagnostics, Germany).

Furthermore, a minute before and after the progressive test, the athletes performed aiming to the target in the standing position with a sporting rifle (20 s). The following variables were recorded (SCATT Shooter Training System, Russia): length of the vertical and horizontal tracking trace (LVTT and LHTT, respectively), maximum target tracking speed (MTTS), and retention time in the area of 9.0 (RT9; Table 2). This shooting system employs a laser aligned with the rifle barrel and an instrumented target to detect the position of the aim point on the target. The system was calibrated for each athlete. The manufacturer guarantees the system accuracy of ±0.1 mm.

On the second day of the examinations, all athletes repeated the incremental treadmill test and aiming test. The tests were identical as on the first day, but they were performed in a normobaric hypoxia chamber at 2000 m (FIO_2_ = 16.6%) to establish aerobic capacity and aiming performance in hypoxia and to determine individual training loads for the IHT sessions (%WR_LT_hyp).

### 2.4. Training Program

**Figure 2:**
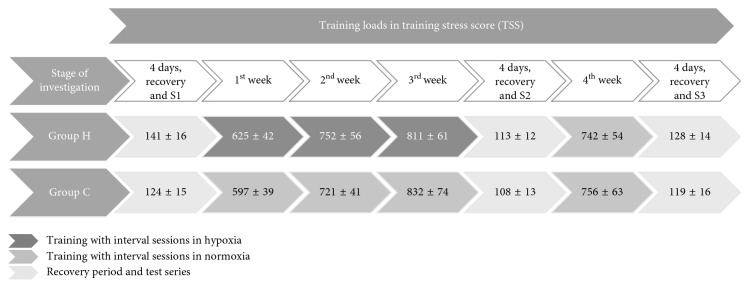
Training loads during investigation in the hypoxia (H) and control (C) groups.

The training program included three microcycles (three weeks) with progressive training loads. The same training program was used for both groups but with different environmental conditions during the selected interval training sessions. Training with lactate threshold intensity was repeated three times a week. The hypoxic group (H) performed training in a normobaric hypoxic chamber (FiO_2_ = 16.6%, equivalent to 2000 m). The normoxic group (C) performed the same training program under normoxic conditions in hypoxic chamber. Both groups were blinded to the environmental conditions during training. Training load was recorded using heart monitor (Forerunner 935, Garmin). It was calculated after each training session and archived using WKO+ 4.0 software (TrainingPeaks, USA) and presented as training stress score (TSS) (Figure 2).

Training intensity was adjusted individually based on the results of lactate threshold determined in normoxia (group C) or hypoxia (group H) during the initial treadmill test. Each workout was performed on a treadmill and consisted of a 15-minute general warm-up (70% of workload at lactate threshold determined in hypoxia and normoxia; WR_LT_hyp/WR_LT_), a 40- to 50-minute main part, and a 5-minute cool-down (60% WR_LT_hyp/WR_LT_). The main part of the workout consisted of four 7 min (1^st^ week), 8 min (2^nd^ week), or 9 min (3^rd^ week) bouts at 100% of WR_LT_hyp/WR_LT_ (group H/group C, respectively) separated by 2 minutes of active recovery. After the warm-up and during the rest between the bouts, the athlete performed aiming to the target in the standing position with a sporting rifle (20 s). Each trial was recorded by SCATT Shooter Training Systems (length of the vertical and horizontal tracking trace, maximum target tracking speed, and retention time in the area of 9.0) whereas aiming beyond the target (the area of 9.0) was alerted by a beep. All parts of the training session (warm-up, main part, and cool-down) and the aiming exercise during recovery bouts in group H were performed in a hypoxic environment.

### 2.5. Statistical Analyses

The results of the study were analyzed by means of the StatSoft Statistica 12.0 software. The results were presented as arithmetic means (*x*) with standard deviations (SD). The statistical significance was set at *p* < 0.05. The Lilliefors test was used to demonstrate the consistency of the results obtained in the study with a normal distribution. The intergroup differences between research series (group × time) were determined using the two-way analysis of variance (ANOVA) for repeated measures. Significance of differences between individual research series in the study groups was calculated based on the post hoc Tukey's test. Effect sizes (ESs) were calculated from standardized differences (Cohen's *d* units). Threshold values for Cohen ES statistics were considered to be small (0.20–0.60), moderate (0.60–1.20), large (1.20–2.0), very large (2.0–4.0), or extremely large (>4.0) [28].

## 3. Results

### 3.1. Aiming Indicators at Rest and after the Incremental Test

Results showed significant group × time interaction for the RT9 at rest, before the incremental test in hypoxia (RT9_rest_, *F* = 6.068, *p* < 0.01). The analysis also revealed a significant group × time interactions for the RT9 and LVTT, after the incremental test in hypoxia (RT9_post_, *F* = 6.102, *p* < 0.01; LVTT_post_, *F* = 3.871, *p* < 0.05) and after the incremental test in normoxia (RT9_post_, *F* = 4.096, *p* < 0.05; LVTT_post_; *F* = 4.609, *p* < 0.05).

After IHT training, RT9_post_ significantly increased by 27.4% (*p* < 0.05; ES: large), whereas LVTT_post_ significantly decreased by 17.1% (*p* < 0.05; ES: large) in normoxic conditions. In the control group, no statistically significant changes were found for aiming variables at rest and postincremental test in normoxia (Table 3). Similar results were obtained for the hypoxic conditions. After IHT intervention, RT9_post_ significantly increased by 26.7% (*p* < 0.001; ES: moderate), whereas LVTT_post_ decreased by 11.3% (*p* < 0.05; ES: very large; Table 4). Furthermore, the post hoc analysis also revealed a significant increase (*p* < 0.05; ES: moderate) in RT9_rest_ in hypoxia for both groups. 14 days after completion of the training program, RT9_rest_, RT9_post_, and LVTT_post_ in both normoxia and hypoxia had returned to initial values.

### 3.2. Aiming Indicators during the First and Last Training Sessions

The analysis of the group × time interaction revealed a significantly different RT9 at rest (RT9_rest_) and RT9 after 1^st^, 2^nd^, 3^rd^, and 4^th^ bout (RT9 1^st^, RT9 2^nd^, RT9 3^rd^, and RT9 4^th^) during the first and last training sessions in normoxia (RT9 1^st^; *F* = 30.09, *p* < 0.001; RT9 4^th^, *F* = 5,036, *p* < 0.05) and hypoxia (RT9_rest_; *F* = 4,05, *p* < 0.067; RT9 2^nd^; *F* = 10,735 *p* < 0.01; RT9 3^rd^*F* = 6,963, *p* < 0.05).

**Figure 3:**
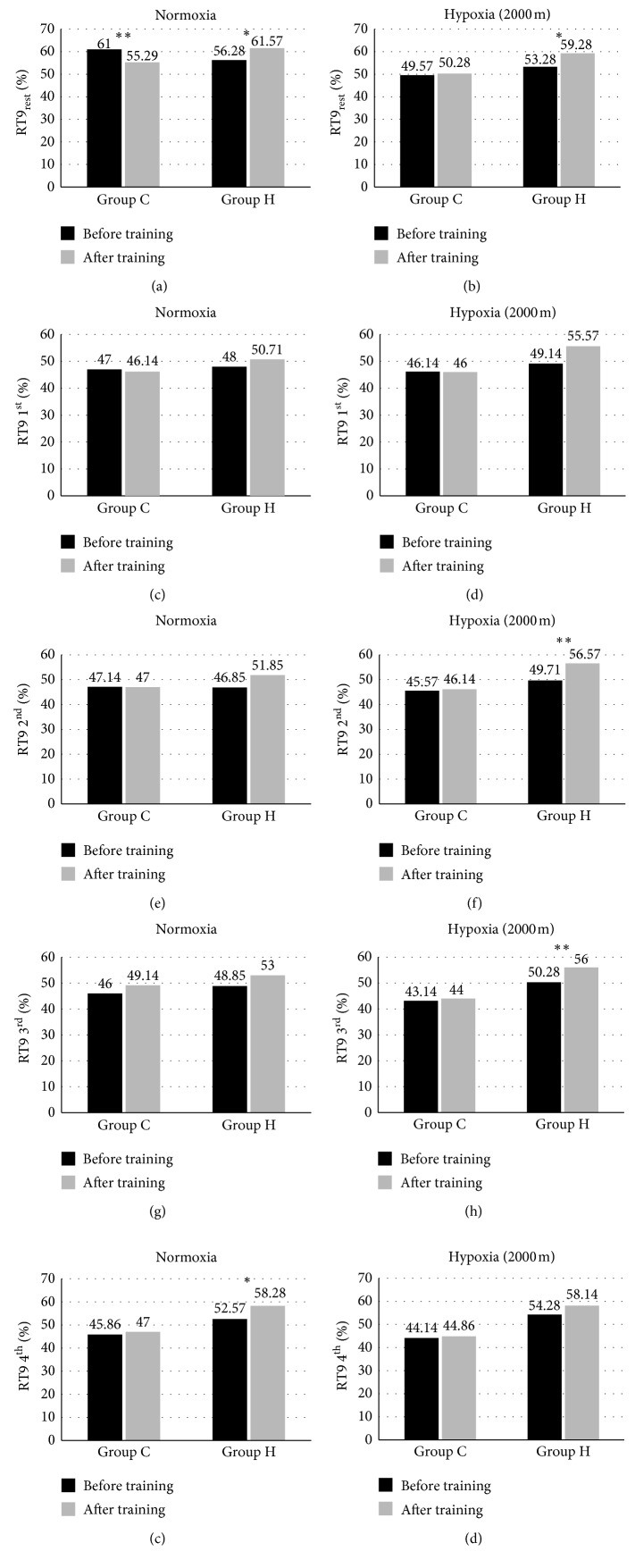
The percent of retention time in the area of 9.0 during 20 s aiming exercise at rest (RT9_rest_) and after 1^st^, 2^nd^, 3^rd^, and 4^th^ bout (RT9 1^st^, RT9 2^nd^, RT9 3^rd^, and RT9 4^th^) in the experimental and control groups (H, *n* = 7; C, *n* = 7) during the first and last training sessions in normoxia and hypoxia (2000 m). ^*∗∗*^*p* < 0.01 indicates statistically significant differences in relation to initial measurements. ^*∗*^*p* < 0.05 indicates statistically significant differences in relation to initial measurements.

After IHT training, RT9_rest_ and RT9 4^th^ during the training session in normoxia significantly increased (*p* < 0.05; ES: moderate) by 9.4% and 10.9%, respectively. On the contrary, in the control group, RT9_rest_ decreased (*p* < 0.01; ES: moderate) by 9.4%. The IHT training also led to significant increases in RT9_rest_ by 11.3% (*p* < 0.05; ES: large), RT9 2^nd^ by 13.8% (*p* < 0.01; ES: large), and RT9 3^rd^ by 11.4% (*p* < 0.01 ES: large) during the training session in hypoxia (Figure 3).

### 3.3. Maximal Workload and Cardiorespiratory Variables

Results showed significant group × time interaction for WR_max_ (*F* = 6.825, *p* < 0.01) and absolute and relative VO_2max_ (*F* = 4.379, *p* < 0.05 and *F* = 5.199, *p* < 0.05, respectively) during the incremental test in normoxia. The analysis also indicated a significantly different RER value (*F* = 6.67, *p* < 0.01) during the incremental test in hypoxia. Furthermore, SO_2capillary_ immediately after the incremental test in hypoxia (SO_2capillary max_) was also significantly different (*F* = 8.42, *p* < 0.01).

**Figure 4:**
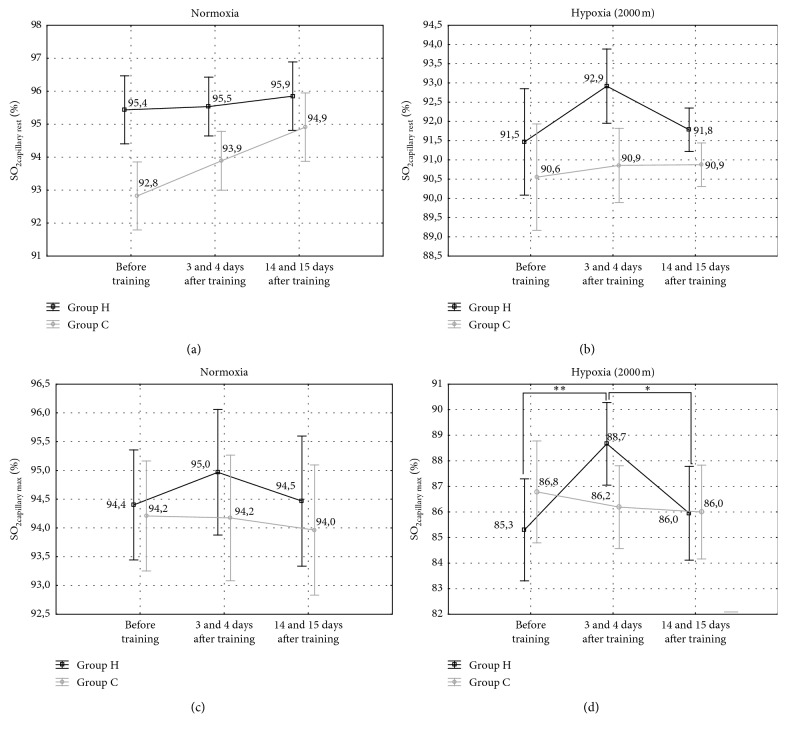
The oxygen saturation at rest (SO_2capillary rest_) and at the end of the incremental test (SO_2capillary max_) in the experimental and control groups (H, *n* = 7; C, *n* = 7) in normoxia and hypoxia (2000 m). ^*∗∗*^*p* < 0.01 indicates statistically significant differences in relation to initial measurements in hypoxia. ^*∗*^*p* < 0.05, indicates statistically significant differences in relation to 3 days post-IHT measurements.

**Figure 5:**
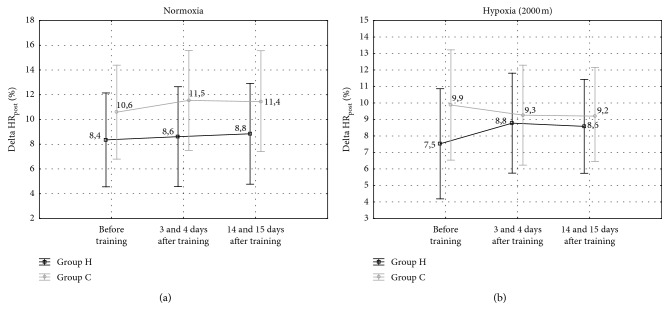
Delta heart rate during 1-minute recovery (delta HR_post_) following the incremental test in the experimental and control groups H, (*n* = 7; C, *n* = 7) in normoxia and hypoxia (2000 m).

The post hoc Tukey's test showed that WR_max_ in normoxia increased significantly (*p* < 0.001; ES: large) by 6.3% after IHT and by 3.6% (*p* < 0.05; ES: moderate) two weeks after IHT compared to the initial measurements (Table 5). The SO_2capillary max_ after the incremental test in hypoxia increased significantly (*p* < 0.001; ES: large) by 4.0% immediately after IHT, but it returned to the pretraining values 14 days after IHT (Figure 4). Additionally, in absolute and relative values of VO_2max_ in normoxia, there was a propensity (*p* < 0.07; ES: moderate) for increasing this value by 5% in group H after IHT (Table 5). Furthermore, in group H, RER value in hypoxia increased significantly (*p* < 0.05; ES: large) by 4.5% immediately after IHT and it remained higher 14 days after IHT in relation to initial measurements (Table 6). Furthermore, there were no significant changes in maximal heart rate (HR_max_) and delta heart rate values during 1-minute recovery (delta HR_post_) following an incremental test in both groups (H and C) in normoxia and hypoxia (Figure 5). Also, there were no significant changes in maximal workload and other cardiorespiratory indices in the C group in normoxia and hypoxia. The training did not have a significant effect on changes in body mass or body composition (Table 7).

## 4. Discussion

### 4.1. Shooting Performance

The high level of technical skill of shooting is a crucial factor for sport success in biathlon. So far, the literature fails to provide information about the effect of hypoxic training on the shooting performance despite the fact that some periods of training and biathlon competitions take place at altitude conditions. Thus, we decided to investigate how 3 weeks of intermittent hypoxic training (IHT) aimed at development of endurance executed with lactate threshold intensity may influence aiming performance of well-trained biathletes.

Our study showed that IHT training has a beneficial effect on biathletes' aiming performance measured by means of shooter training systems. Importantly, the improvement of aiming efficacy was observed both in normoxia and in normobaric hypoxia at rest, in the postincremental test, and during training sessions. These improvements were related first and foremost to enhanced retention time in the target. After IHT, RT9_post_ increased by 27.4% in normoxia and 26.7% in hypoxia compared to the pretest. Furthermore, postexercise length of the vertical tracking trace was reduced by 17.1% in normoxia and 11.3% in hypoxia. No significant changes in this variable were found in the group that trained in normoxia. Analysis of aiming bouts performed in the first and last training sessions also demonstrated that IHT has a positive impact on RT9 during aiming bouts performed after exercise at LT intensity. The hypoxic stimulus was a significant factor that has an impact on the improvement in RT9, since improvements in this variable were not found in the control group although the group followed the same training program in normoxia.

The results of our study indicate the improvement in terms of factors responsible for aiming performance after IHT training, which does not occur after identical training in normoxia. We suppose that reduction of aim-point fluctuation that we recorded in our study can result from improved stability of hold and postural balance, which are important technical components in shooting accuracy in the standing position [2, 35–37]. The study conducted by Sattlecker et al. [38] indicated that in the case of the load with physical exercise, body sway in the standing position and vertical rifle motion in the lying position determine the shooting performance in biathletes. Shooting ability can be disturbed by involuntary movements such as physiological tremor. However, with such a form of fast shooting used in biathlon, tremor size plays an insignificant role, since initial preprogrammed impulse is more important than the sensory control phase [39]. Additionally, weapon mass and inertia in biathlon mitigate the increase in tremor size, and the method of gripping the weapon reduces the transmission of high-frequency tremor [39]. Moreover, isometric effort that participates to a larger extent in maintaining a standing shooting posture is also associated with a brief reduction in tremor size [40].

Some studies have indicated that shooting performance depends on the intensity of the exercise which precedes the shooting task [1, 41] and it is less effective after completion of exercise at higher intensity. In practice, biathletes, when approaching the shooting range, attempt to reduce HR, which is aimed at improving shooting accuracy [4]. In our study, we did not observe any changes in HR_max_ and delta HR_post_ in normoxia and hypoxia after IHT. However, HR_max_ values were achieved at a higher maximum workload after IHT, which may have had an effect on delta HR_post_. This observation and the results of our previous research [42], where after the IHT training we noted a significant reduction in HR_avg_ during cycling time trial, accompanied by an increase in average power, suggest that IHT may affect the reduction of submaximal HR and thus contribute to the improvement of the shooting performance. However, in opposition to the above postulate, some researchers argue that lower HR during shooting is not necessarily conducive to the improvements in the shooting performance. Lower HR allows for a longer duration of weapon stabilization before shooting. However, as HR declines, stroke volume (SV) increases, leading to greater body motion and rifle deflection and, consequently, higher likelihood of a mistake. Therefore, faster reduction in HR before shooting is not as beneficial as it would seem [39]. The relative importance of HR and SV to optimal shooting performance still remains unclear.

Shooting performance can be disturbed not only following physical exercise but also after hypoxic exposure. Obviously, depending on the level of its intensity, exposure to hypoxia can lead to higher muscle tremor, disturbed balance, and deteriorated postural stability as well as reduced ability to perform psychomotor activities or weakening of cognitive functions [22, 43–46]. These changes can have a negative effect on the shooting performance in biathlon when competing at altitude. So far, Tharion et al. [47] reported deteriorated aiming accuracy, whereas Kryskow et al. [26] demonstrated a decline in shooting speed after exposure to the altitude of 4300 m. Furthermore, Muza et al. [48] found that the shooting performance was significantly reduced already at an altitude of 2743 m. Important examinations of the effect of hypoxia on the shooting performance were performed by Moore et al. [23]. The study examined the shooting performance at rest and following exercise at sea level and altitudes of 1000–4000 m. The shooting score was significantly lower at an altitude of 4000 m compared to other altitudes. A downward propensity in shooting scores was found at an altitude of 3000 m. No decline in shooting performance was demonstrated at altitudes of 1000 m and 2000 m. Due to the very low number of studies concerning this problem, it remains unclear whether there is a specific hypoxia threshold which has an impact on reduction in shooting performance.

There are a few studies that have demonstrated that although acute hypoxia may impair shooting performance, a stay at altitude leads to adaptive changes and restoration of shooting performance to standards reached at sea level [47, 48]. Our study was the first to reveal the impact of IHT training on aiming performance of biathletes either in hypoxic or in normoxic environments. One of the mechanisms behind the improvement of aiming abilities after IHT training can be an increase in SO_2capillary_. It is worth noting that in our study, improvements in postexercise aiming performance were accompanied by an increase in postexercise SO_2capillary_, whereas deterioration in performance was associated with a decline in SO_2capillary_ (Figure 4). SO_2capillary_ after the incremental test in hypoxia was 4% higher following the IHT protocol compared to the values recorded before the experiment, and at 2 weeks after completion of hypoxia training, it had returned to the initial level. A similar propensity was found for postexercise SO_2capillary_ in normoxia. This observation is consistent with findings published by Moore et al. [23] and Kryskow et al. [26], who found a significant correlation between SO_2_ and shooting performance in hypoxia. Moore et al. [23] suggested that in hypoxia, the correlations of shooting accuracy with SO_2_ and ventilation rate (VR) are critical to shooting performance. Kryskow et al. [26] observed a correlation between SO_2_ and shooting speed, whereas Moore et al. [23] found correlations of SO_2_ and VR with marksmanship. Since both the increase in altitude and physical exercise lead to a decline in SO_2_ and increase in VR, exercise performed in hypoxia translates into a reduction in shooting performance [23].

### 4.2. Aerobic Capacity

In recent years, the vast majority of research investigating the application of altitude/hypoxic training has focused on aerobic endurance outcomes [24]. It is suggested that endurance athletes could benefit from IHT (with intensity around the anaerobic threshold), especially during the precompetitive phase [49]. The present study extends our understanding of this issue in the situation where IHT was implemented in the second mesocycle of the preparation period. Biathletes are characterized by decreased endurance capacity during this period compared to the competition period. Therefore, the main objective of the training process during this period is the reestablishment of endurance capacity. Therefore, we proposed the lactate threshold intensity during an interval workout, which is the one of the most effective methods to improve endurance performance. Despite this, the results did not demonstrate a meaningful improvement in the analyzed cardiorespiratory variables after the training program. However, the significant increase in WR_max_ as well as the propensity (*p* < 0.07) for the increase in VO_2max_ values was only observed following the IHT training.

Previous reports on the effectiveness of IHT protocols in improvement of aerobic performance remain unclear. There are reports which have demonstrated an increase in aerobic capacity and exercise capacity following the IHT protocols [42, 50–54]. However, part of the data indicate that the benefits of the IHT training are similar to the effects of training in normoxia, whereas hypoxia does not represent an additional stimulus for greater adaptive changes [29, 55–60]. Although Hendriksen and Meeuwsen [56] found a significant increase in maximal power output (W_max_) following 10 days of training in hypoxia (2500 m), Roels et al. [59] demonstrated a significant increase in peak power output (PPO) after 3 weeks of the IHT protocol (3000 m), and Morton and Cable [58] observed significant improvements in W_max_ and VO_2max_ after 4 weeks of IHT (2750 m); these changes did not differ significantly from the findings in groups that trained in normoxia. Similarly, in the experiment conducted by Geiser et al. [55] a 6-week IHT protocol (3850 m) yielded improvements in both VO_2max_ and PPO. However, significant differences were not found between IHT effects and training in normoxia. It is worth noting that despite the lack of statistical significance, the change in VO_2max_ after IHT was 2% greater than after training at sea level. As suggested by Levine and Stray-Gundersen [61] and Fulco et al. [62], who reviewed investigations with elite athletes in whom training adaptations were difficult to improve due to many years of training, even small improvements (documented as statistically insignificant during the examinations) in one of the capacity indices may translate into improved exercise capacity and improved competitive performance.

The lack of IHT training efficiency in terms of aerobic capacity can be partially explained by an improperly chosen load during training sessions in hypoxia. As shown in the findings of a meta-analysis performed by Bonetti and Hopkins [63], exercise intensity can be critical to adaptive changes during training in hypoxia. Our previous examinations in a group of cyclists [42, 53] demonstrated that the IHT protocol based on prolonged exercise (30 to 40 min) at lactate threshold is an important training resource for the improvement in VO_2max_ and exercise capacity at sea level. In the present study, although the length of a single repetition during the interval training ranged from 7 to 9 minutes, total exercise volume at the level of 100% WR_LT_hyp ranged from 28 to 36 min and was similar to the volume used in our previous experiments. This training protocol led to a significant increase in final load (WR_max_) during exercise to exhaustion, whereas a noticeable increasing propensity (by 5%) was observed in VO_2max_. The lack of significant changes in this area is most likely to result from the small research sample. The results obtained in our study are similar to the results presented by Dufour et al. [50] and Zoll et al. [51] where, after interval training in hypoxia (3000 m) with similar intensity and total exercise volume to those used in our study (24–40 min at the second ventilatory threshold), an improvement of 5% VO_2max_ was observed.

### 4.3. Study Limitations and Perspectives

To the best of our knowledge, our study is the first in which the impact of IHT training on the aiming efficiency was assessed in hypoxia and normoxia. Our investigations, however, are not without certain limitations. Firstly, we conducted a study on small sample size. The small sample size resulted from limited access to a larger number of biathletes at the appropriate sport skill level. Future research will be planned with the participation of biathletes from other national teams in order to verify the results obtained in our study. Secondly, in our study, we found that the improvement of steadiness of aiming after IHT training was accompanied by a significant increase in postexercise SO_2capillary_. In addition to the SO_2_ level, ventilation rate (VR) measurements would also be recommended to perform during shooting test. Determining the level of postexercise rating of perceived exertion (RPE) would also be desirable. This would allow for evaluation of the relationship between these indicators and shooting performance, especially in hypoxic conditions. Establishing the above dependences could partly explain the mechanisms responsible for improving shooting performance after IHT training.

This is the first study in this area; therefore, many questions remain open, such as the following: Does IHT training in normobaric hypoxia also affect the shooting performance in hypobaric hypoxia (terrestrial hypoxia)? Does the improvement of steadiness of aiming after the IHT training, which was recorded in our study, directly translate into shooting performance determined by the number of hits? Is the decrease in aim-point fluctuation after IHT training associated with the improvement of postural balance, rifle stability, or both factors? These and many other issues require further research.

## 5. Conclusions

The results obtained in our study demonstrated unequivocally an influence of IHT training on aiming performance. Following a three-week IHT protocol, adaptations to hypoxia were observed, leading to improved resting and postexercise steadiness of aiming, irrespective of the environmental conditions (i.e., in normoxia or hypoxia). Furthermore, the study demonstrated a beneficial effect of the IHT protocol on exercise capacity of biathletes. These results confirm the usefulness of hypoxic training in preparation of biathletes, not only in the context of improved exercise capacity but also enhanced aiming performance.

The results have practical implications. Although the experiment was carried out during the preparation period, we suggest that the proposed IHT protocol can also be used successfully during the competitive period. The use of IHT does not require the acclimation phase, often associated with deterioration of mood and the need for reducing the training load. Consequently, IHT is not associated with the risk of a decrease in the fitness level, which is particularly unwanted in the competitive period. Additionally, the benefits in the form of improved shooting performance following IHT training may have a significant effect on the sports performance, especially in competitions held at altitude.

## Figures and Tables

**Table 1:** Characteristics of study participants.

Variable	Group H (*n* = 7)	Group C (*n* = 7)
Age (years)	21.2 ± 1.5	20.1 ± 2.0
Training experience (years)	7.1 ± 1.5	6.5 ± 1.1
Body height (cm)	179.8 ± 4.7	180.2 ± 3.1
Body mass (kg)	73.2 ± 4.2	71.1 ± 5.1
Fat content (%)	9.8 ± 4.1	10.4 ± 2.7
VO_2max_ (ml/kg/min)	63.6 ± 4.8	65.7 ± 1.5

**Table 2:** Variables indicating aim-point fluctuation.

Variable	Description
RT9 (%)	Percent of retention time in the area of 9.0 during 20 s aiming test. Greater RT9 values indicate better steadiness of aiming.

LHTT (mm)	Length of the horizontal tracking trace.
	Smaller LHTT values indicate decrease in aim-point fluctuation in the *X* (horizontal) axis.

LVTT (mm)	Length of the vertical tracking trace.
	Smaller LVTT values indicate decrease in aim-point fluctuation in the *Z* (vertical) axis.

MTTS (mm/s)	Maximum target tracking speed.
	Smaller MTTS values indicate decrease in aim-point fluctuation.

**Table 3:** Mean values selected shooting indicators recorded in the experimental and control groups (H, *n* = 7; C, *n* = 7) at rest and postincremental test in normoxia.

Variable	Group H			Cohen's *d*			Group C			Cohen's *d*			ANOVA		
	S1_N_	S2_N_	S3_N_	*d*_1_	*d*_2_	*d*_3_	S1_N_	S2_N_	S3_N_	*d*_1_	*d*_2_	*d*_3_	Time	Group	Interaction
RT9_rest_ (%)	63.28 ± 8.51	66.42 ± 11.50	63.00 ± 9.72	0.34	0.35	0.03	61.00 ± 6.61	60.85 ± 7.69	59.00 ± 6.20	0.02	0.29	0.35	*F* = 2.21; *p*=0.13	*F* = 0.84; *p*=0.37	*F* = 0.85; *p*=0.44

RT9_post_ (%)	41.28 ± 9.77	**52.60** ± **9.28**^*∗*^	43.28 ± 10.59	1.23	0.96	0.29	38.42 ± 6.34	37.85 ± 2.26	37.00 ± 6.44	0.13	0.19	0.24	*F* = 3.97; *p*=0.032	*F* = 4.86; *p*=0.047	*F* = 4.09; *p*=0.029

LVTT_rest_ (mm)	62.28 ± 9.91	59.85 ± 10.41	57.71 ± 14.51	0.26	0.18	0.40	70.71 ± 16.19	68.14 ± 10.42	82.14 ± 22.70	0.20	0.85	0.63	*F* = 0.89; *p*=0.42	*F* = 5.24; *p* = 0.04	*F* = 2.16; *p*=0.13

LVTT_post_ (mm)	100.71 ± 10.17	**83.50** ^*∗*^ ± **11.44**	98.57 ± 6.26	1.71	1.76	0.27	103.57 ± 24.61	107.00 ± 13.97	101.42 ± 114.92	0.47	0.26	0.21	*F* = 2.29; *p*=0.12	*F* = 2.87; *p*=0.11	*F* = 4.61; *p*=0.018

LHTT_rest_ (mm)	69.71 ± 15.98	64.42 ± 8.40	66.14 ± 10.60	0.45	0.19	0.28	77.42 ± 12.16	80.57 ± 13.98	83.00 ± 15.43	0.26	0.18	0.43	*F* = 0.17; *p*=0.84	*F* = 5.74; *p*=0.03	*F* = 1.04; *p*=0.37

LHTT_post_ (mm)	96.57 ± 19.93	92.28 ± 13.16	96.42 ± 14.35	0.27	0.32	0.01	89.42 ± 11.70	90.28 ± 8.38	93.42 ± 12.08	0.09	0.33	0.36	*F* = 0.89; *p*=0.42	*F* = 0.37; *p*=0.55	*F* = 0.50; *p*=0.61

MTTS_rest_ (mm/s)	196.42 ± 26.88	179.42 ± 17.71	187.85 ± 29.13	0.81	0.38	0.33	186.00 ± 10.26	188.57 ± 16.76	191.28 ± 35.72	0.20	0.11	0.22	*F* = 0.72; *p*=0.49	*F* = 0.004; *p*=0.94	*F* = 1.28; *p*=0.30

MTTS_post_ (mm/s)	250.71 ± 62.07	215.71 ± 56.78	247.00 ± 30.52	0.65	0.78	0.06	244.42 ± 46.94	241.57 ± 15.07	248.57 ± 25.93	0.09	0.36	0.12	*F* = 1.48; *p*=0.24	*F* = 0.16; *p*=0.69	*F* = 0.86; *p*=0.43

Test series in normoxia: S1_N_, before training; S2_N_, 3 and 4 days after training; S3_N_, 14 and 15 days after training. Effect size: *d*_1_, between S1_N_ and S2_N_; *d*_2_, between S2_N_ and S3_N_; *d*_3_, between S1_N_ and S3_N_. ^*∗*^*p* < 0.05, statistically significant differences in relation to initial measurements (S1_N_). RT9, percent of retention time in the area of 9.0 during 20 s aiming test; LVTT, length of the vertical tracking trace; LHTT, length of the horizontal tracking trace; MTTS, maximum target tracking speed; rest, at rest; post, postexercise.

**Table 4:** Mean values of selected shooting indicators recorded in the experimental and control groups (H, *n* = 7; C, *n* = 7) at rest and postincremental test in hypoxia (2000 m).

Variable	Group H			Cohen's *d*			Group C			Cohen's *d*			ANOVA		
	S1_N_	S2_N_	S3_N_	*d*_1_	*d*_2_	*d*_3_	S1_N_	S2_N_	S3_N_	*d*_1_	*d*_2_	*d*_3_	Time	Group	Interaction
RT9_rest_ (%)	57.42 ± 9.62	**65.71** ± **7.36**^*∗*^	**60.14** ± **6.33**^#^	1.04	0.88	0.36	54.42 ± 4.19	**57.80** ± **3.60**^*∗*^	55.40 ± 2.63	0.94	0.82	0.31	*F* = 35.83; *p* < 0.0001	*F* = 2.68; *p*=0.12	*F* = 6.07; *p*=0.007

RT9_post_ (%)	39.57 ± 7.72	**50.14** ± **11.94**^*∗*^	43.28 ± 8.65	1.13	0.71	0.49	38.85 ± 4.37	39.42 ± 4.75	37.85 ± 7.15	0.14	0.28	0.18	*F* = 8.23; *p*=0.002	*F* = 2.11; *p*=0.17	*F* = 6.10; *p*=0.007

LVTT_rest_ (mm)	64.42 ± 17.43	59.00 ± 8.38	64.28 ± 7.29	0.43	0.73	0.01	64.00 ± 7.85	64.57 ± 12.63	66.42 ± 3.99	0.06	0.21	0.42	*F* = 0.78; *p*=0.46	*F* = 0.29; *p*=0.60	*F* = 0.52; *p*=0.59

LVTT_post_ (mm)	106.14 ± 6.59	**94.14** ± **5.92**^*∗*^	**103.50** ^#^ ± **17.20**	2.07	0.79	0.21	114.28 ± 10.96	112.85 ± 7.49	111.14 ± 7.42	0.16	0.20	0.31	*F* = 4.46; *p*=0.02	*F* = 5.22; *p*=0.04	*F* = 3.87; *p*=0.034

LHTT_rest_ (mm)	75.75 ± 21.88	68.14 ± 11.06	70.71 ± 12.72	0.46	0.23	0.29	77.42 ± 15.26	74.85 ± 7.91	76.28 ± 11.11	0.23	0.16	0.09	*F* = 1.61; *p*=0.22	*F* = 0.48; *p*=0.49	*F* = 0.41; *p*=0.66

LHTT_post_ (mm)	89.42 ± 16.45	77.74 ± 16.52	93.71 ± 17.93	0.77	1.00	0.27	107.42 ± 11.45	110.42 ± 12.47	117.42 ± 11.31	0.27	0.63	0.95	*F* = 3.70; *p*=0.04	*F* = 16.78; *p*=0.001	*F* = 1.51; *p*=0.24

MTTS_rest_ (mm/s)	193.57 ± 11.80	172.85 ± 15.77	183.85 ± 18.55	1.61	0.69	0.67	186.00 ± 10.26	188.57 ± 16.76	191.28 ± 35.72	0.24	0.52	0.20	*F* = 5.96; *p*=0.008	*F* = 4.97; *p*=0.045	*F* = 1.96; *p*=0.19

MTTS_post_ (mm/s)	281.42 ± 71.51	253.57 ± 61.82	266.42 ± 39.44	0.45	0.27	0.28	291.42 ± 33.30	288.57 ± 37.04	290.71 ± 30.33	0.09	0.07	0.02	*F* = 0.94; *p*=0.40	*F* = 1.07; *p*=0.32	*F* = 0.63; *p*=0.54

Test series in hypoxia: S1_H_, before training; S2_H_, 3 and 4 days after training; S3_H_, 14 and 15 days after training. Effect size: *d*_1_, between S1_H_ and S2_H_; *d*_2_, between S2_H_ and S3_H_; *d*_3_, between S1_H_ and S3_H_. ^*∗*^*p* < 0.05, statistically significant differences in relation to initial measurements (S1_H_). ^#^*p* < 0.05, statistically significant differences in relation to 3 days post-IHT measurements (S2_H_). RT9, percent of retention time in the area of 9.0 during 20 s aiming test; LVTT, length of the vertical tracking trace; LHTT, length of the horizontal tracking trace; MTTS, maximum target tracking speed; rest, at rest; post, postexercise.

**Table 5:** Mean values of maximal workload and selected cardiorespiratory indices recorded in the experimental and control groups (H, *n* = 7; C, *n* = 7) during the incremental test in normoxia.

Variable	Group H			Cohen's *d*			Group C			Cohen's *d*			ANOVA		
	S1_N_	S2_N_	S3_N_	*d*_1_	*d*_2_	*d*_3_	S1_N_	S2_N_	S3_N_	*d*_1_	*d*_2_	*d*_3_	Time	Group	Interaction
WR_max_ (W)	372.14 ± 14.72	**395.57** ^*∗*^ ± **17.57**	**385.42** ^*∗*^ ± **13.22**	1.56	0.70	1.03	361.71 ± 26.06	369.40 ± 23.27	354.28 ± 32.42	0.34	0.58	0.27	*F* = 16.01; *p* < 0.0001	*F* = 3.91; *p*=0.07	*F* = 6.82; *p*=0.005

VO_2max_ (l/min)	4646.71 ± 271.83	**4885.57** ^**§**^ ± **314.47**	4762.42 ± 420.04	0.88	0.36	0.35	4686.28 ± 413.80	4614.85 ± 372.69	4523.00 ± 498.84	0.20	0.23	0.38	*F* = 1.91; *p*=0.17	*F* = 0.63; *p*=0.44	*F* = 4.38; *p*=0.02

VO_2max_ (ml/kg/min)	63.57 ± 4.79	**67.00** ^**§**^ ± **5.65**	66.71 ± 6.75	0.71	0.05	0.58	65.71 ± 1.49	65.14 ± 2.19	63.57 ± 2.69	0.33	0.69	1.06	*F* = 1.43; *p*=0.25	*F* = 0.20; *p*=0.66	*F* = 5.20; *p*=0.013

VE_max_ (l/min)	163.18 ± 16.60	168.50 ± 13.22	164.57 ± 18.32	0.38	0.27	0.09	156.72 ± 8.56	163.57 ± 12.59	153.50 ± 11.27	0.69	0.91	0.35	*F* = 2.67; *p*=0.09	*F* = 1.40; *p*=0.26	*F* = 0.47; *p*=0.62

RER_max_	1.10 ± 0.04	1.14 ± 0.05	1.10 ± 0.03	0.99	1.03	0.12	1.10 ± 0.02	1.16 ± 0.03	1.10 ± 0.05	2.03	1.27	0.00	*F* = 14.61; *p*=0.0001	*F* = 0.45; *p*=0.51	*F* = 0.11; *p*=0.89

HR_max_ (bpm)	193.42 ± 6.29	192.28 ± 7.60	189.00 ± 7.75	0.18	0.47	0.69	200.57 ± 4.96	199.00 ± 6.92	196.14 ± 4.52	0.28	0.53	1.01	*F* = 6.33; *p*=0.006	*F* = 5.08; *p*=0.043	*F* = 0.02; *p*=0.098

Test series in normoxia: S1_N_, before training; S2_N_, 3 and 4 days after training; S3_N_, 14 and 15 days after training. Effect size: *d*_1_, between S1_N_ and S2_N_; *d*_2_, between S2_N_ and S3_N_; *d*_3_, between S1_N_ and S3_N._^*∗*^*p* < 0.05, statistically significant differences in relation to initial measurements (S1_N_). ^§^*p* < 0.07, propensity in relation to initial measurements (S1_N_). WR_max_, maximal workload during the incremental test; VO_2max_, maximal oxygen uptake; VE_max_, maximal ventilation; RER_max_, maximal respiratory ratio during the incremental test; HR_max_, maximal heart rate.

**Table 6:** Mean values of maximal workload and selected cardiorespiratory indices recorded in the experimental and control groups (H, *n* = 7; C, *n* = 7) during the incremental test in hypoxia (2000 m).

Variable	Group H			Cohen's *d*			Group C			Cohen's *d*			ANOVA		
	S1_N_	S2_N_	S3_N_	*d*_1_	*d*_2_	*d*_3_	S1_N_	S2_N_	S3_N_	*d*_1_	*d*_2_	*d*_3_	Time	Group	Interaction
WR_max_ (W)	347.85 ± 19.10	350.71 ± 16.97	349.42 ± 18.84	0.17	0.08	0.09	312.00 ± 26.08	314.14 ± 24.40	317.71 ± 22.35	0.09	0.16	0.25	*F* = 0.87; *p*=0.43	*F* = 9.88; *p*=0.008	*F* = 0.43; *p*=0.65

VO_2max_ (l/min)	4162.00 ± 328.59	4250.42 ± 204.20	4199.14 ± 241.02	0.35	0.25	0.14	3894.71 ± 428.52	3918.57 ± 407.45	4056.14 ± 450.52	0.06	0.35	0.40	*F* = 2.35; *p*=0.12	*F* = 1.83; *p*=0.20	*F* = 2.18; *p*=0.13

VO_2max_ (ml/kg/min)	56.83 ± 5.77	58.83 ± 4.58	57.83 ± 4.62	0.42	0.18	0.26	54.42 ± 4.07	55.14 ± 3.80	57.42 ± 2.29	0.20	0.79	0.98	*F* = 3.97; *p*=0.033	*F* = 1.20; *p*=0.30	*F* = 2.08; *p*=0.15

VE_max_ (l/min)	167.17 ± 15.15	166.14 ± 17.99	171.41 ± 18.45	0.27	0.31	0.07	153.80 ± 10.46	155.57 ± 10.41	152.21 ± 9.25	0.18	0.37	0.17	*F* = 2.51; *p*=0.10	*F* = 3.99; *p*=0.07	*F* = 0.21; *p*=0.81

RER_max_	1.10 ± 0.05	**1.15** ^*∗*^ ± **0.04**	**1.15** ^*∗*^ ± **0.02**	1.23	0.14	1.31	1.15 ± 0.03	1.14 ± 0.02	1.12 ± 0.02	0.13	1.02	0.99	*F* = 2.43; *p*=0.11	*F* = 0.11; *p*=0.75	*F* = 6.67; *p*=0.005

HR_max_ (bpm)	189.71 ± 5.96	187.85 ± 6.20	186.14 ± 7.55	0.33	0.27	0.57	193.42 ± 4.54	193.71 ± 4.95	191.42 ± 4.68	0.06	0.51	0.47	*F* = 4.17; *p*=0.03	*F* = 3.02; *p*=0.11	*F* = 0.62; *p*=0.54

Test series in hypoxia: S1_H_, before training; S2_H_, 3 and 4 days after training; S3_H_, 14 and 15 days after training. Effect size: *d*_1_, between S1_H_ and S2_H_; *d*_2_, between S2_H_ and S3_H_; *d*_3_, between S1_H_ and S3_H_. ^*∗*^*p* < 0.05, statistically significant differences in relation to initial measurements (S1_H_). WR_max_, maximal workload during the incremental test; VO_2max_, maximal oxygen uptake; VE_max_, maximal ventilation; RER_max_, maximal respiratory ratio during the incremental test; HR_max_, maximal heart rate.

**Table 7:** Mean values of body mass and percent of body fat in the experimental (H, *n* = 7) and control groups (C, *n* = 7) during investigation.

Variable	Group H			Cohen's *d*			Group C			Cohen's *d*			ANOVA		
	S1	S2	S3	*d*_1_	*d*_2_	*d*_3_	S1	S2	S3	*d*_1_	*d*_2_	*d*_3_	Time	Group	Interaction
BM (kg)	73.2 ± 4.2	71.7 ± 3.2	72.4 ± 3.6	0.41	0.20	0.22	71.1 ± 5.1	70.7 ± 4.3	70.9 ± 5.5	0.09	0.04	0.04	*F* = 3.51; *p*=0.045	*F* = 0.41; *p*=0.53	*F* = 1.13; *p*=0.34
% fat	9.8 ± 4.1	9.8 ± 3.6	9.6 ± 3.9	0.01	0.07	0.08	10.4 ± 2.7	10.5 ± 1.9	9.2 ± 0.84	0.03	0.83	0.62	*F* = 3.06; *p*=0.07	*F* = 0.03; *p*=0.86	*F* = 1.31; *p*=0.28

S1, before training; S2, 3 days after training; S3, 14 days after training. Effect size: *d*_1_, between S1 and S2; *d*_2_, between S2 and S3; *d*_3_, between S1 and S3. BM—body mass; % fat—percent of body fat.

## Data Availability

The results data used to support the findings of this study are available from the corresponding author upon request.
